# Clinical features and treatment outcome of lymphoepithelioma-like carcinoma from multiple primary sites: a population-based, multicentre, real-world study

**DOI:** 10.1186/s12890-022-02097-6

**Published:** 2022-09-22

**Authors:** Meiting Chen, Yungchang Chen, Xiaojie Fang, Zhao Wang, Xingxiang Pu, Chaoyong Liang, Hongqiang Guo, Qian Li, Fei Pan, Huangming Hong, He Huang, Jiman Li, Tongyu Lin

**Affiliations:** 1 Department of Medical Oncology, Sun Yat-Sen University Cancer Center, State Key Laboratory of Oncology in South China, Collaborative Innovation Center for Cancer Medicine, No. 651, Dongfeng East Road, Yuexiu District, Guangzhou, 510060 China; 2 Senior Ward/ Phase I Clinical Trial Ward, Sichuan Cancer Hospital & Institue, Sichuan Cancer Center, School of Medicine, University of Electronic Science and Technology of China, Chengdu, 610041 China; 3 Department of Thoracic Medical Oncology, Hunan Cancer Hospital, The Affiliated Cancer Hospital of Xiangya School of Medicine, Central South University, Yuelu District, 283 Tongzipo Road, Changsha, 410013 Hunan China; 4 Department of Medical Oncology, Guangxi Medical University Cancer Hospital, Nanning, 530021 Guangxi Zhuang Autonomous Region China; 5 The Affiliated Cancer Hospital of Zhengzhou University, Henan Cancer Hospital, Zhengzhou, China; 6 Department of Pathology, Sichuan Cancer Hospital & Institue, Sichuan Cancer Center, School of Medicine, University of Electronic Science and Technology of China, Chengdu, 610041 China

**Keywords:** Lymphoepithelioma-like carcinoma, Rare cancer, Treatment pattern, Survival, Multicentre study

## Abstract

**Background:**

Lymphoepithelioma-like carcinoma (LELC) is a rare and unique subtype of cancer that histologically resembles undifferentiated nasopharyngeal carcinoma (NPC). The population-based analysis of LELC and the optimal treatment remains unclear.

**Materials and methods:**

This real-world, retrospective study investigated 770 patients with LELC for primary site, treatment, and survival outcomes from 2005 to 2019 from five cancer centres in China. The overall survival (OS) of different subgroups was appraised by log-rank tests and Kaplan–Meier analysis.

**Results:**

Primary sites LELC included the lung (597 cases, 77.5%), salivary gland (115 cases, 14.9%), and others. The median progression-free survival (PFS) of LELC patients was 47.4 months. The median overall survival (OS) was not reached. The 5-year survival rate for LELC patients was 77.8%. Most patients in stages I and II received surgery. The majority of patients in stage III received surgery and radiotherapy. More than half of the patients in stage IV received chemotherapy. Among relapsed or metastatic cases receiving chemotherapy, patients who received immunotherapy at any time presented with a superior OS than those without immunotherapy (*P* < 0.0001, HR = 0.39, 95% CI 0.25–0.63). Compared with the SEER database, patients with LELC had a better prognosis than NPC, with a 5-year overall survival of 77.3% vs. 56.8% (*P* < 0.001).

**Conclusion:**

Our data provide treatment patterns and outcomes for LELC from various primary sites. Randomized controlled studies are necessary to further define the standard of care for patients with LELC.

*Trial registration* This clinical trial was registered at ClinicalTrials.gov (No. NCT04614818).

**Supplementary Information:**

The online version contains supplementary material available at 10.1186/s12890-022-02097-6.

## Introduction

Lymphoepithelioma-like carcinoma (LELC), an Epstein-Barr virus (EBV)-driven cancer, resemble nasopharynx carcinoma but can originate from the pharyngeal and other foregut-derived organs, such as the lung and gastrointestinal tract [1]. The 2004 World Health Organization classification previously recognized LELC of the lung as a subtype of large cell carcinoma, whereas the current 2015 World Health Organization classification scheme categorizes LELC under other and unclassified carcinomas. The lung is the most common primary site of LELC, and some retrospective studies have summarized cases of pulmonary LELC [2]. Several case reports have focused on LELC from different primary sites, such as parotid glands, submandibular glands, stomach, breast, skin, and liver [3–5].

Since LELC is a rare type of cancer, its optimal management is not clear. Total resection of tumours seems to be the proper treatment for early-stage LELC in the salivary glands, liver, and thymus [5–7]. Lin and colleagues reported that gemcitabine-based chemotherapy and palliative thoracic radiotherapy were associated with a better overall survival in 127 irresectable pulmonary LELC patients from a single centre [8]. Although several studies have shown high programmed death legand-1 (PD-L1) expression in LELCs [6, 9–12], its response to immunotherapy is controversial in different reports [13, 14]. The genome landscape of LELC revealed a similarity to NPC [9, 12, 15], but comparisons of the survival and prognosis of LELC and NPC are lacking. Analysis of the characteristics, treatment, survival, and prognosis of LELC is warranted. Thus, we collected data from multiple centres to explore the optimal treatment pattern of LELC and summarized the prognosis of LELC compared to NPC.

## Materials and methods

### Patient selection

From August 2005 to September 2019, we enrolled patients with LELC at five cancer centres, including Sun Yat-sen Cancer Centre (SYSUCC), Sichuan Cancer Centre, Henan Cancer Hospital, Hunan Cancer Hospital and Guangxi Medical University Cancer Hospital. Eligible patients had histologically confirmed LELC and had clear medical records. Their medical records were analysed for investigation. All patients underwent treatment selected by experienced doctors at cancer centres. The study protocol was approved by the ethical committee of the Sun Yat-sen University Cancer Centre (approval number B2020–289–01). This clinical trial was registered at ClinicalTrials.gov (No. NCT04614818) since 4th, November, 2020.

To compare the prognosis of LELC with NPC and pulmonary squamous carcinoma, two cohorts were established from the Surveillance, Epidemiology, and End Results (SEER) database. The patient data from 2010 to 2016 were acquired by SEER ∗ Stat software (Version 8.3.5, http://seer.cancer.gov/seerstat/download). The inclusion criteria for the NPC and lung cancer cohort were as follows: (1) a clear pathological diagnosis of NPC and squamous cell carcinoma of the lung, respectively; (2) a diagnosis from 2010 to 2016; (3) the TNM stage of the patients followed the seventh edition of the AJCC; and (4) a clear survival time. The exclusion criteria in both cohorts were cases with only autopsy or death certificates and invalid follow-up. The data cut-off was at March 12, 2021.

### Parameters

The following clinical and laboratory data were collected: age, gender, smoking history, Eastern Cooperative Oncology Group performance status (ECOG PS), metastatic sites, clinical stage, treatment modality, EBV DNA, genome mutation status, and ALK rearrangement status. The detection for EBV infection was demonstrated by serum EBV-DNA levels. EBER in situ hybridization was exanimated as routine pathology process. Patient follow-ups were obtained through medical records or by telephone interview. Efficacy was assessed according to Response Evaluation Criteria in Solid Tumours (RECIST) version 1.1 [16].

For the SEER database, the demographic data included age (0–24, 25–49, 50–74, and ≥ 75 years), gender (male, female), and race (white, black, and others (American Indian/Alaska Native or Asian/Pacific Islander)). The clinical properties incorporated T stage (T0, T1, T2, T3, T4, and unknown), N stage (N0, N1, N2, N3, and unknown), and organ metastases (none, yes, and unknown).

### Statistical analysis

The study population for all analyses included patients enrolled in the study who had an adequate baseline tumour assessment. Descriptive statistics were used to summarize the patient characteristics, treatment administration, and objective response, and the results are presented as medians and ranges. Survival was measured from the initiation of therapy until death. Overall survival (OS) was defined as the time between the initiation of treatment to death from any cause. Patients who did not die were censored at the date of last contact. The date of censoring data was at March 12, 2021. The objective response rate (ORR), disease control rate (DCR), disease-free survival (DFS) and progression-free survival (PFS) were also analysed. Pearson chi-square or Fisher’s exact test and Wilcoxon tests were used to identify between-group differences for categorical variables and continuous variables, respectively. Univariable Cox regression analyses and multivariable proportional hazards regression models were carried out to identify independent prognostic factors. All reported P values were two-sided, and *P* < 0.05 was considered to be statistically significant. All statistical analyses were carried out using SPSS version 25 (SPSS Inc., Chicago, IL, USA) and R version 4.0.2.

## Results

### Baseline character

**Fig. 1:**
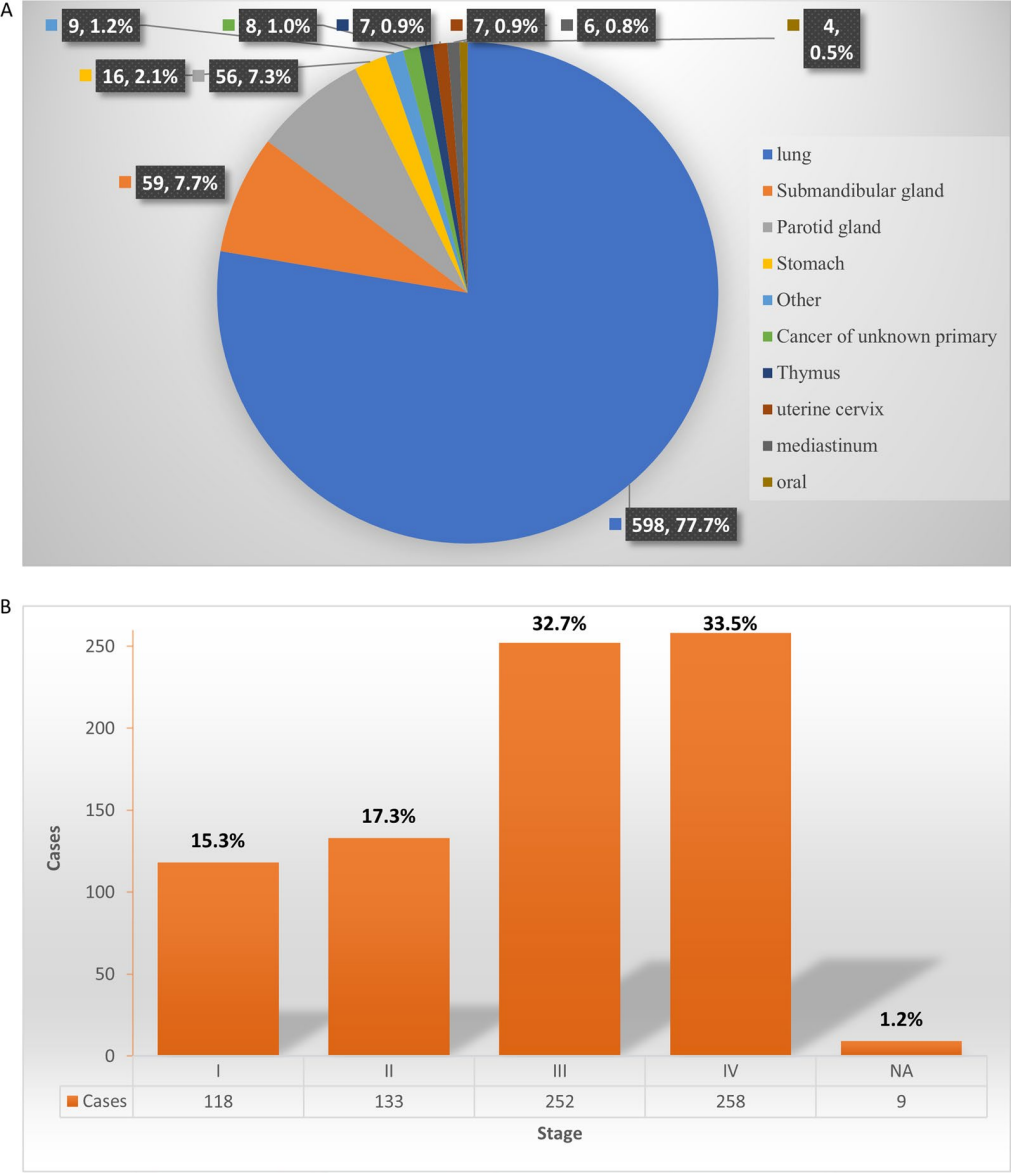
The distribution of origin of LELC (**A**) and initial staging of LELC (**B**)

In our study, 831 cases of LELC were collected, 15 patients did not receive any treatment. Three hundred patient follow-ups were obtained by telephone interview and 470 patients followed through clinic interview. As of the data cut-off, 46 patients were lost to follow-up. Finally, a total of 770 consecutive patients were included in the analyses. 117 (15.2%) patients died, and 653 (84.8%) were alive. The median follow-up time was 34.6 months (range 0.3–179 months). The baseline characteristics of the patients are summarized in Table 1. Among the 770 patients, the median age was 52 years (range, 12–82), 408 (53.0%) were female, 721 (93.6%) had an ECOG PS of 0, and 618 (80.3%) were never-smokers. The origin of the LELCs is summarized in Fig. 1A. The majority of patients had LELC of lung origin (77.7%). A total of 115 patients (14.9%) had the primary site at the salivary gland, and 16 patients (2.2%) were diagnosed with gastric LELC. The other uncommon primary sites (less than 1%) included the thymus, uterine cervix, mediastinum, oropharynx, oesophagus, liver, tonsil, ovary, penis, and external auditory canal. Eight patients were diagnosed with LELC of unknown primary source.

**Table 1:** Patient characteristics

Characteristics	Cases (n, %)
Age [median (range), years]	52(12–82)
Gender	
Male	362(47.0%)
Female	408(53.0%)
Smokers	152(19.7%)
Primary site	
Lung	598(77.7%)
Salivary glands	115(14.9%)
GI tract	19(2.5%)
others	38(4.9%)
Stage at diagnosis	
I	118(15.3%)
II	133(13.3%)
III	252(32.7%)
IV	258(33.5%)
NA	9(1.2%)
Initial treatment	
Surgery	522(67.8%)
Chemotherapy	311(40.4%)
Radiotherapy	100(13.0%)
CK	
Positive	414(53.8%)
Negative	6(0.8%)
NA	350(45.4%)
p63	
Positive	524(68.1%)
Negative	17(2.2%)
NA	229(29.7%)
CK5/6	
Positive	555(72.1%)
Negative	18(2.4%)
NA	197(25.6%)
EBER	
Positive	668(98.4%)
Negative	11(1.6%)
NA	91
EBV-DNA copy	
≥ 10^3^ IU/mL	174(50.6%)
< 10^3^ IU/mL	53(15.4%)
0	117(34.0%)
NA	426
Gene sequencing	
EGFR mutation	10(3.3%)
ALK arrangement	3(1.0%)
TP53 mutation	7(2.3%)
NOTCH mutation	2(0.7%)
No driver gene mutation detected	283(92.8%)
NA	465

In 679 cases with EBER results, 98.4% of them was EBER positive. A total of 344 (44.7%) patients had baseline tests for EBV-DNA, and 174 (50.6%) showed EBV DNA copy numbers higher than 10^3^ copy/mL. A total of 305 (39.6%) patients underwent genetic tests, including EGFR mutations and ALK fusion. EGFR mutations were detected in only 10 (3.3%) patients. ALK mutations were detected in only 3 (1.0%). Seven cases presented with a mutation of TP53. Thirty-four patients had PD-L1 expression detected, and 16 patients had PD-L1 expression greater than 50%. 25 patients (73.5%) presented PD-L1 expression higher than 1%. Most patients were at stage III (32.7%) or IV (33.5%) at the time of initial diagnosis (Fig. 1B).

### Survival outcome

**Fig. 2:**
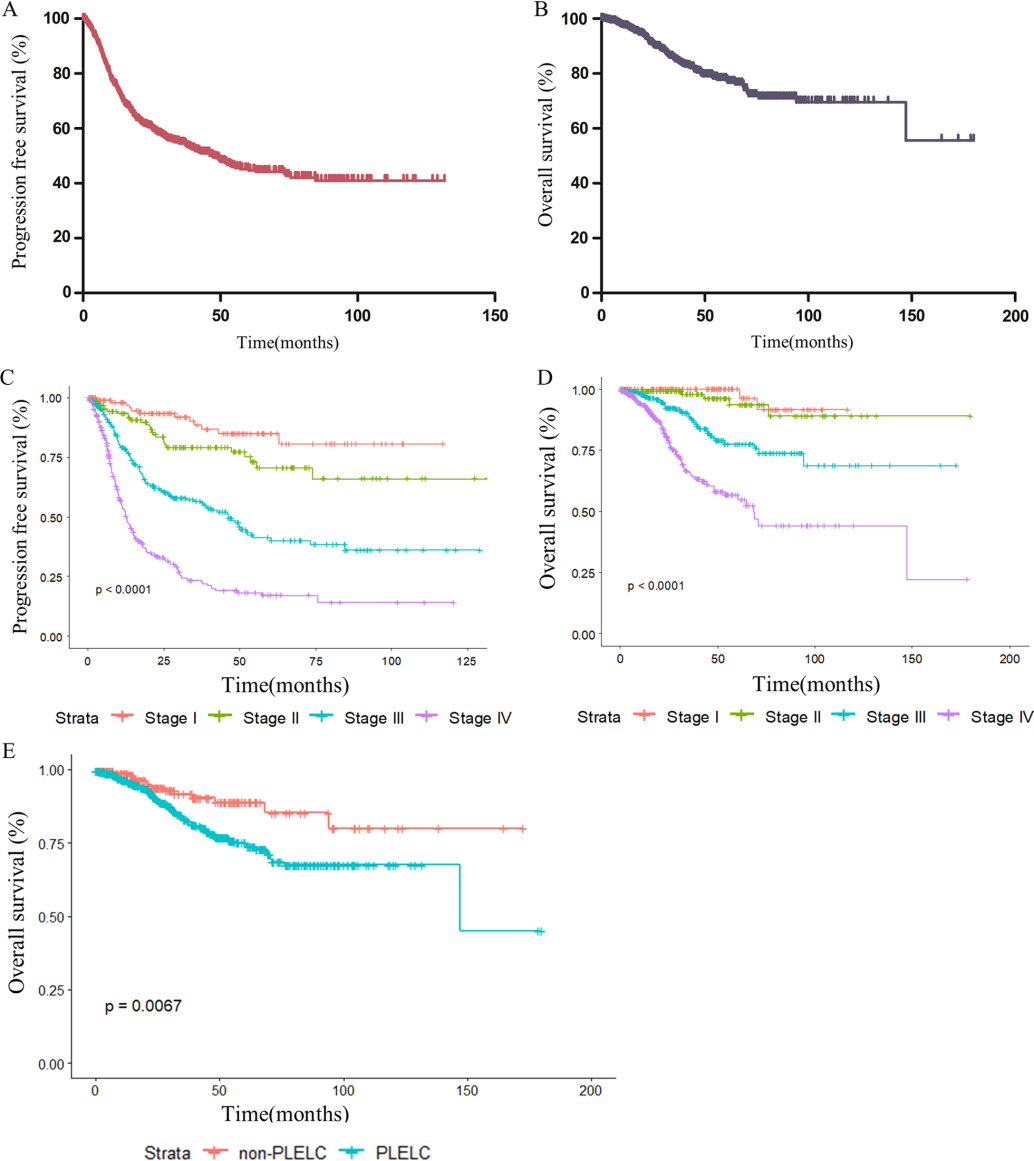
PFS (**A**) and OS (**B**) in total LELC patients. PFS (**C**) and OS (**D**) for LELC patients according to different stage. OS for LELC patients according to different primary site (**E**)

**Fig. 3:**
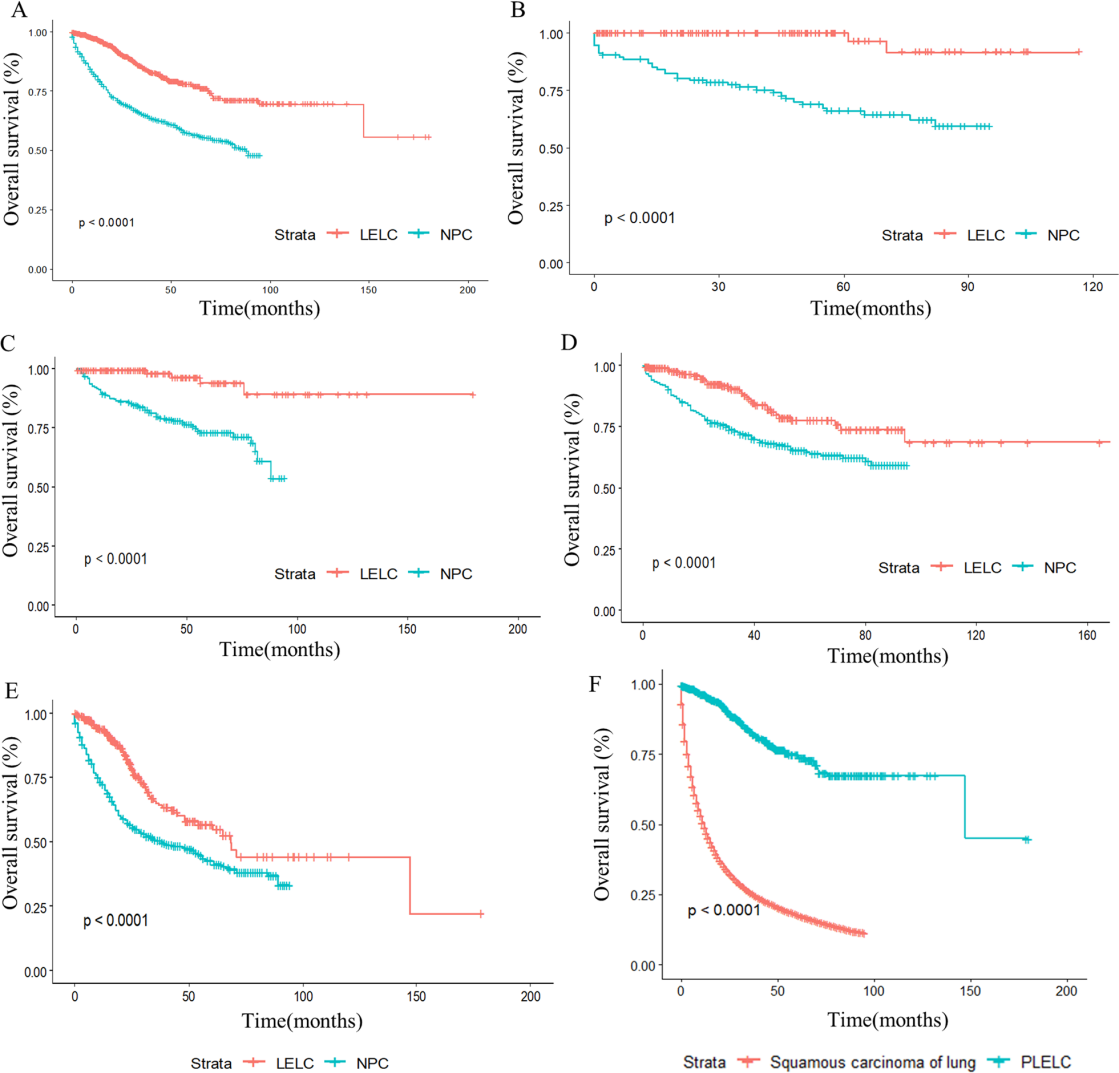
OS compared LELC with NPC in seer data base. Survival for total patients (**A**) and according to patients at stage I (**B**), II (**C**), III (**D**) and IV (**E**). OS compared PLELC with squamous carcinoma of lungs in seer data base (**F**)

The PFS and OS of all LELC patients are shown in Fig. 2A and B, respectively. The median PFS in LELC patients was 47.4 months, and the median OS was not reached. The survival analysis among the different stages and primary sites is shown in Fig. 2C, D and E, respectively, resulting in PLELC presenting worse outcomes than any other primary site. Compared with NPC from the SEER database, LELC showed a better OS (Fig. 3A), and subgroup analysis for stage I, II, III and IV LELC revealed the same results compared with NPC at stage I, II, III and IV, respectively (Fig. 3 B, C, D and E). When comparing pulmonary LELC with squamous carcinoma of the lung, it was found that pulmonary LELC also showed superior OS than squamous cell carcinoma of the lung (Fig. 3F).

### Treatment pattern for patients at stage I–II

**Fig. 4:**
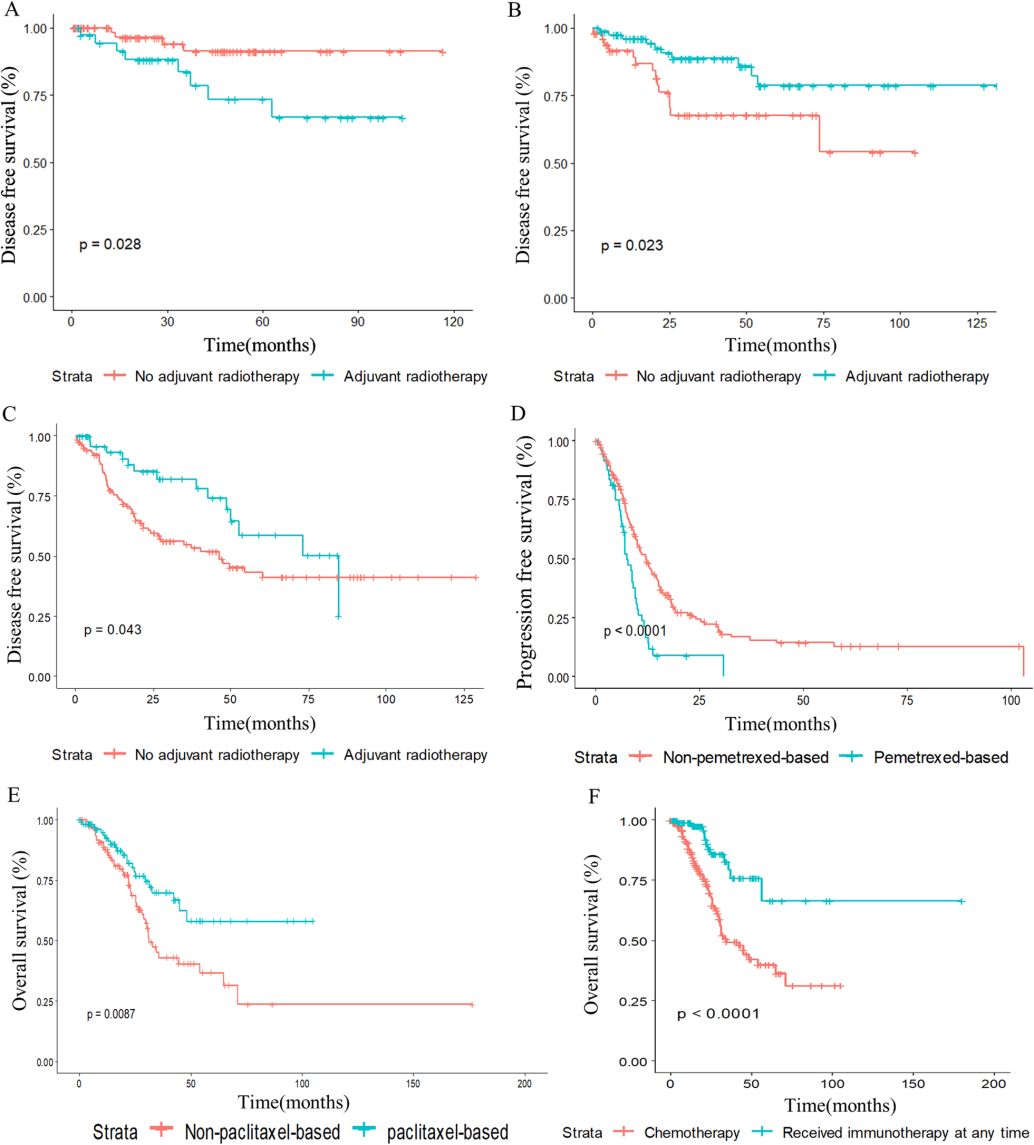
DFS in patients receiving adjuvant chemotherapy at stage I (**A**) and II (**B**). DFS in patients receiving adjuvant radiotherapy at stage III (**C**). PFS (**D**) and OS (**E**) in patients receiving pemetrexed-based chemotherapy as first-line regimen at stage IV or after relapsed. OS in patients at stage IV or after relapsed receiving immunotherapy at any time (**F**)

The treatment pattern for patients at different stages varied. The survival of LELC patients at different stages is shown in Fig. 2D. For 118 patients in stage I, all patients underwent surgery. Among them, 5 patients received neoadjuvant chemotherapy before surgery. Thirty-eight patients received adjuvant chemotherapy, and 9 patients received adjuvant radiotherapy. Patients receiving adjuvant chemotherapy showed inferior DFS (*P* = 0.028, HR = 3.77, 95% CI 1.15–12.3) and a similar OS to those without adjuvant chemotherapy (Fig. 4A and Additional file 1: Fig. S1A). Due to limited number of patients received radiotherapy at stage I, the role of radiotherapy was uncertain (Additional file 1: Fig. S1B, C). For 133 patients in stage II, 131 patients received surgery, while one patient with oesophageal LELC received sequencing chemotherapy and radiotherapy, and one patient with PLELC received concurrent chemotherapy and radiotherapy. Seventeen patients received neoadjuvant chemotherapy. Eighty patients received adjuvant chemotherapy, and 20 patients received adjuvant radiotherapy. Patients receiving adjuvant chemotherapy showed a longer DFS (*P* = 0.023, HR = 0.38, 95% CI 0.16–0.87) but similar OS to those without adjuvant chemotherapy (Fig. 4B and Additional file 2: Fig. S2A). No significant difference was observed between patients with and without adjuvant radiotherapy (Additional file 2: Fig. S2B, C).

### Treatment pattern for patients at stage III

For 252 patients in stage III, 169 patients underwent surgery, among whom 24 patients received neoadjuvant chemotherapy. In 169 patients who underwent surgery, 121 patients received adjuvant therapy, with 107 receiving chemotherapy and 50 radiotherapies. Patients unsuitable for surgery received chemotherapy (n = 28) and radiotherapy (n = 55) based on the evaluation case by case of experienced physicians. The median PFS in stage III patients was 46.1 months, but the median OS was not reached. Patients receiving adjuvant chemotherapy showed similar OS rates to those without adjuvant chemotherapy (Additional file 3: Fig. S3A, B). However, patients receiving adjuvant radiotherapy showed a longer DFS (*P* = 0.043, HR = 0.59, 95% CI 0.35–0.98) but a similar OS (Fig. 4C and Additional file 3: Fig. S3C).

### Treatment pattern for patients at stage IV

For 258 patients in stage IV at initial diagnosis, 133 patients received first-line chemotherapy, and 87 patients received surgery; 35 patients received radiotherapy; 3 patients received targeted therapy including osimertinib, afatinib and alectinib; 179 patients relapsed after surgery or radiotherapy, and 69 of them received chemotherapy as first-line treatment. For 226 patients treated with different chemotherapy regimens, it was demonstrated that most patients received a platinum-based regimen as first-line chemotherapy. The ORR and DCR for all regimens were 46.9% and 83.2%, respectively. According to the different regimens, 48 patients received pemetrexed- and platinum-based regimens, 33 patients received gemcitabine- and platinum-based regimens, and the majority of patients (n = 110) received paclitaxel- and platinum-based regimens. The median PFS in stage IV patients was 12.5 months, and the median OS was 68.4 months. Comparing the different regimens, pemetrexed-based regimens had a worse PFS and OS (Fig. 4D, E). The paclitaxel-based regimen showed a superior OS but similar PFS (Additional file 4: Fig. S4A, B). However, gemcitabine-based regimens presented better PFS but a similar OS (Additional file 4: Fig. S4C, D). In addition to chemotherapy, some patients were also treated with antiangiogenic therapy, such as bevacizumab, anti-EGFR antibodies and immune checkpoint inhibitors. Seventeen patients received chemotherapy combined with antiangiogenic regimens, including bevacizumab, recombinant human endostatin, apatinib and nilotinib. Eight patients received chemotherapy combined with anti-EGFR antibodies, including cetuximab and nituzumab. For patients with and without anti-angiogenesis or anti-EGFR, no significant difference was shown in their survival outcome (Additional file 5: Fig. S5A, B).

Ninety-six patients received immune checkpoint inhibitors, including PD-1 antibody and PD-L1 antibody. Thirty-two patients received immunotherapy as first-line treatment. Among relapsed or metastatic cases receiving chemotherapy, patients who received immunotherapy at any time with metastasis or relapses presented a superior OS than those without immunotherapy (*P* < 0.0001, HR = 0.39, 95% CI 0.25–0.63, Fig. 4F). No significant difference in OS was found between patients receiving immunotherapy as first-line or after prior chemotherapy. For patients at stage IV, those who had received radiotherapy showed better outcomes than those who had not received radiotherapy (Additional file 4: Fig. S4E).

### Prognosis predictors

For the prognosis of LELC patients, it was found that the following clinical factors significantly predicted poor survival in univariable analysis: ECOG PS ≥ 2, stage III-IV, lung as the primary site, bone metastasis, liver metastasis, pleura metastasis, nonregional lymph node metastasis, adrenal metastasis, and a high level of serum EBV DNA copy (Additional file 6: Fig. S6). Multivariable analysis demonstrated that five variables had a negative prognostic influence on OS, including ECOG PS ≥ 2, stage III–IV, a high level of EBV-DNA, liver metastasis and bone metastasis (Table 2).

**Table 2:** Univariate and multivariate analyses of potential prognostic factors for OS

Characteristics	Univariate analysis		Multivariate analysis	
	HR (95% CI)	*P* value	HR (95% CI)	*P* value
Age				
< 60				
≥ 60	1.141(0.736–1.768)	0.555	0.969(0.617–1.525)	0.894
Gender				
Male				
Female	1.025(0.976–1.482)	0.896	1.015(0.655–1.572)	0.947
ECOG score				
< 2				
≥ 2	3.828(2.331–6.287)	< 0.0001***	1.753(1.031–2.980)	0.038*
Stages				
I–II				
III–IV	9.152(4.257–19.67)	< 0.0001***	5.560(2.510–12.34)	< 0.0001***
Lung origin	2.144(1.225–3.753)	0.0076**	1.581(0.862–2.899)	0.138
Lung metastasis	3.062(1.885–4.973)	< 0.0001***	0.852(0.490–1.480)	0.569
Bone metastasis	4.761(3.178–7.132)	< 0.0001***	1.793(1.107–2.903)	0.0176*
Liver metastasis	7.145(4.715–10.83)	< 0.0001***	2.591(1.537–4.369)	0.0003***
Pleura metastasis	3.639(2.409–5.495)	< 0.0001***	0.948(0.569–1.580)	0.838
Non-regional lymph node metastasis	4.789(3.124–7.341)	< 0.0001***	1.642(0.968–2.783)	0.065
Adrenal metastasis	3.989 (1.625–9.79)	0.0025**	0.774(0.292–2.054)	0.607
Smoker	1.087(0.688–1.718)	0.72	0.936(0.541–1.619)	0.814
High EBV DNA copy	3.750(2.585–5.441)	< 0.0001***	1.726(1.134–2.626)	0.011*

*HR* hazard ratio, *CI* confidence interval, *ECOG* Eastern Cooperative Oncology Group, *EBV* Epstein-Barr virus

## Discussion

The understanding for LELC was generally based on single centre study for PLELC, LELC of salivary glands [2, 15, 17]. The rare origin of LELC included thyroid, skin, thymus, liver, breast, external auditory canal, lips, laryngopharyngeal, urinary tract, and tonsil mainly reported by a few cases [5, 18–26]. The clinicopathological characteristics and prognosis of LELC from other origins based on multiple centres and large sample sizes are unknown.

In our study, we reported the clinicopathological characteristics, treatment pattern and prognosis of LELC from different origins at multiple cancer centres, with the largest sample size of LELC so far. In summary, we reported the treatment pattern of 770 LELC cases from five cancer centres. It was demonstrated that LELC presented better survival outcomes than NPC patients. The benefit of adjuvant chemotherapy and radiotherapy varied among different stages of LELC. For advanced disease, the pemetrexed-based regimen showed inferior PFS and OS. Patients receiving immunotherapy at any time with metastasis or relapses presented a superior OS than those without immunotherapy. Radiotherapy was also beneficial for patients at an advanced stage.

The pathological characteristics among different primary sites of LELC were fairly consistent and histologically resembled undifferentiated NPC. Most cases of LELC were closely related to EBV infection and were EBER-positive [1, 3, 11, 27, 28], but EBER was negative in most cases of LELC from the head and neck skin [18, 23]. In our study, p40, CK5/6, p63 and EBER were positive in most patients. More than half of the patients (174 out of 344 cases detected EBV-DNA) revealed a high level of baseline serum EBV DNA. High EBV-DNA was also associated with poor survival. EBV DNA should be monitored during treatment and follow-up. In a multivariate model of 127 advanced PLELC cases, cycles of first-line chemotherapy, palliative thoracic radiotherapy, and baseline EBV DNA were significantly associated with OS [8]. Chen B and colleagues also showed that positive serum EBV-DNA appeared to be a predictor of PFS [29]. In our study, high EBV DNA was correlated with poor survival, which is consistent with a previous study [8, 29]. It was also concluded that ECOG PS ≥ 2, stage III–IV, liver metastasis and bone metastasis independently predicted survival in LELC patients. Thus, the evaluation of liver and bone as well as serum EBV DNA is important for patients with LELC at the time of initial diagnosis.

In our study, compared with NPC in the SEER database, LELC showed a superior prognosis. The reason might be that most patients at an early stage had opportunities for radical surgery. Several case reports have shown that surgical resection is the basic treatment for LELC of the breast, liver, thyroid, thymus, and skin [4, 11, 18, 21]. The analysis from the SEER database of 179 cases of salivary gland LELC demonstrated that surgery and postsurgical radiotherapy significantly improved OS [30]. For adjuvant therapy, patients in stage I did not benefit from adjuvant chemotherapy or radiotherapy. Although adjuvant chemotherapy prolonged DFS, OS was not improved. The survival outcome varied among patients at stage III. For patients receiving surgery at stage III, adjuvant radiotherapy improved DFS. In our study, neither adjuvant chemotherapy nor radiotherapy improved OS. Thus, more prospective exploration of the application of adjuvant therapy is necessary.

The sensitivity to radiotherapy and chemotherapy in LELC is not clear. Most patients diagnosed with parotid LELC receive radiotherapy [7, 30], and concurrent chemoradiotherapy is effective for patients with middle ear and sinonasal LELC [26, 31]. The prognosis for patients receiving surgery seems to be based on the primary site. The recurrence rate for liver LELC is much lower than that for hepatic cell carcinoma and intrahepatic cholangiocarcinoma [3, 5]. However, patients with thymic LELC relapse rapidly after surgery [4, 6]. In our study, 141 out of 383 patients relapsed after surgery. The treatment for patients in an advanced stage or after a relapse needs further exploration. In our study, the patients receiving radiotherapy had a superior OS. First-line chemotherapy was selected based on experience in different cancer centers. In a study including 127 patients with advanced PLELC, paclitaxel and platinum-based chemotherapy presented a median PFS of 12 months, while pemetrexed-based regimens only achieved a median PFS of 5 months [8]. Our study included 226 patients receiving first-line chemotherapy and demonstrated that the pemetrexed-based regimen showed a poor PFS and OS. However, the benefit of TP and GP regimens is controversial. GP revealed a prolonged PFS but a similar OS, while TP presented the opposite result. The optimal regimen for LELC and the role of radiotherapy should be further explored in prospective studies. Multimodality treatment is also warranted for LELC.

The majority of pulmonary LELCs (74.3%–75.8%) overexpressed PD-L1 [10, 32]. High expression of PD-L1 was also detected in LELC of the thymus and liver [6, 33]. However, the efficacy of immunotherapy in LELC remains unclear. Some case reports presented a promising response to PD-L1 blockade [34]. However, other cases revealed rapid progression after immunotherapy [14]. In our study, patients receiving immunotherapy at any time showed a prolonged OS, which suggested an important role of immune checkpoint inhibitors. Similar to NPC, the genomic landscape of LELC for the lung and liver showed a high frequency of TP53 mutations [9, 12, 15]. However, mutation analysis suggested a low tumour burden and low levels of driver gene mutations in PLELC patients [15, 32, 35]. The mechanism by which patients respond or are resistant to PD-1/PD-L1 blockade needs further understanding. In our study, one patient with advanced PLELC treated with pembrolizumab and paclitaxel-based chemotherapy progressed after six cycles and harbored secondary amplification of PI3KCA and IL-7R, which might be one possible mechanism for PD-1 resistance [14]. In our study, NGS analysis and PD-L1 expression were only detected in a few patients. Among patients detected PD-L1, 73.5% of them presented PD-L1 overexpression high than 1%. More biomarkers to predict the outcome of immunotherapy are under exploration. Anti-EGFR antibody and anti-angiogenesis therapy play an important role in NPC and lung cancer, respectively. However, no significant difference was shown in patients receiving either anti-EGFR antibody or anti-angiogenesis therapy. Thus, the role of anti-EGFR and antiangiogenic agents needs more exploration.

The major limitation of this study lies in its retrospective nature and its heterogeneity in baseline risk and treatment factors, which may have led to potential bias. In outr study, the number of patients with PD-L1 detection and NGS results was limited, which made it difficult to understand the relation between efficacy of immunotherapy and biomarkers. Our further research might focus on PD-L1 expression and NGS in available samples. Nonetheless, these findings add to the growing body of data demonstrating the clinicopathological and treatment patterns in patients with LELC from many kinds of primary sites. The main strength of the present study is that it is the first report summarizing LELC from different origins, including a large number of real-world patients; thus, these findings might be generalizable to a broad population of patients. Therefore, prospective clinical trials to confirm the optimal treatment for LELC are warranted.

## Conclusion

LELC from various primary sites resembles NPC but with better survival outcomes. The benefit of adjuvant therapy for LELC at early stage varied. The immunotherapy and radiotherapy showed better survival outcome for advanced LELC patients. The optional treatment for different stage of LELC needed more prospective studies.

## Supplementary Information

**Additional file 1. Figure S1:** Kaplan–Meier survival analysis for OS in patients receiving adjuvant chemotherapy at stage I (A). DFS(B) and OS(C) in patients receiving adjuvant radiotherapy at stage I

**Additional file 2. Figure S2:** Kaplan–Meier survival analysis for OS in patients receiving adjuvant chemotherapy at stage II (A). DFS(B) and OS(C) in patients receiving adjuvant radiotherapy at stage II

Additional file 3 Supplement Figure 3 Kaplan–Meier survival analysis for OS in patients receiving adjuvant radiotherapy at stage III (A). DFS(B) and OS(C) in patients receiving adjuvant chemotherapy at stage III

**Additional file 4. Figure S4:** Kaplan–Meier survival analysis for PFS (A) and OS (B) in patients receiving paclitaxel-based chemotherapy as first-line regimen at stage IV or after relapsed. PFS (C) and OS (D) in patients receiving gemcitabine-based chemotherapy as first-line regimen at stage IV or after relapsed. OS in patients at stage IV or after relapsed receiving radiotherapy (E)

**Additional file 5. Figure S5:** Kaplan–Meier survival analysis for OS in patients receiving anti- angiogenesis (A) and anti- EGFR (B) therapy at stage IV or after relapsed. Kaplan–Meier survival analysis for PFS in patients receiving anti-angiogenesis (C) and anti-EGFR (D) therapy at stage IV or after relapsed

**Additional file 6. Figure S6:** Kaplan–Meier survival analysis for OS according to ECOG PS≥2 (A), stage III-IV (B) , high level of EBV-DNA (C), liver metastasis (D) and bone metastasis (E)

## Data Availability

The datasets used and/or analysed during the current study available from the corresponding author on reasonable request.

## Abbreviations

LELC: Lymphoepithelioma-like carcinoma
NPC: Nasopharyngeal carcinoma
PFS: Progression-free survival
OS: Overall survival
EBV: Epstein-Barr virus
PD-L1: Programmed death legand-1
SEER: Surveillance, Epidemiology, and End Results
ECOG PS: Eastern Cooperative Oncology Group performance status
RECIST: Response Evaluation Criteria in Solid Tumours
ORR: Objective response rate
DCR: Disease control rate
DFS: Disease-free survival
