# Supplementary figures and images for: Clinical features and treatment outcome of lymphoepithelioma-like carcinoma from multiple primary sites: a population-based, multicentre, real-world study
Source: BMC Pulm Med. 2022 Sep 22;22:360. doi: 10.1186/s12890-022-02097-6 (PMC9494884; doi:10.1186/s12890-022-02097-6)

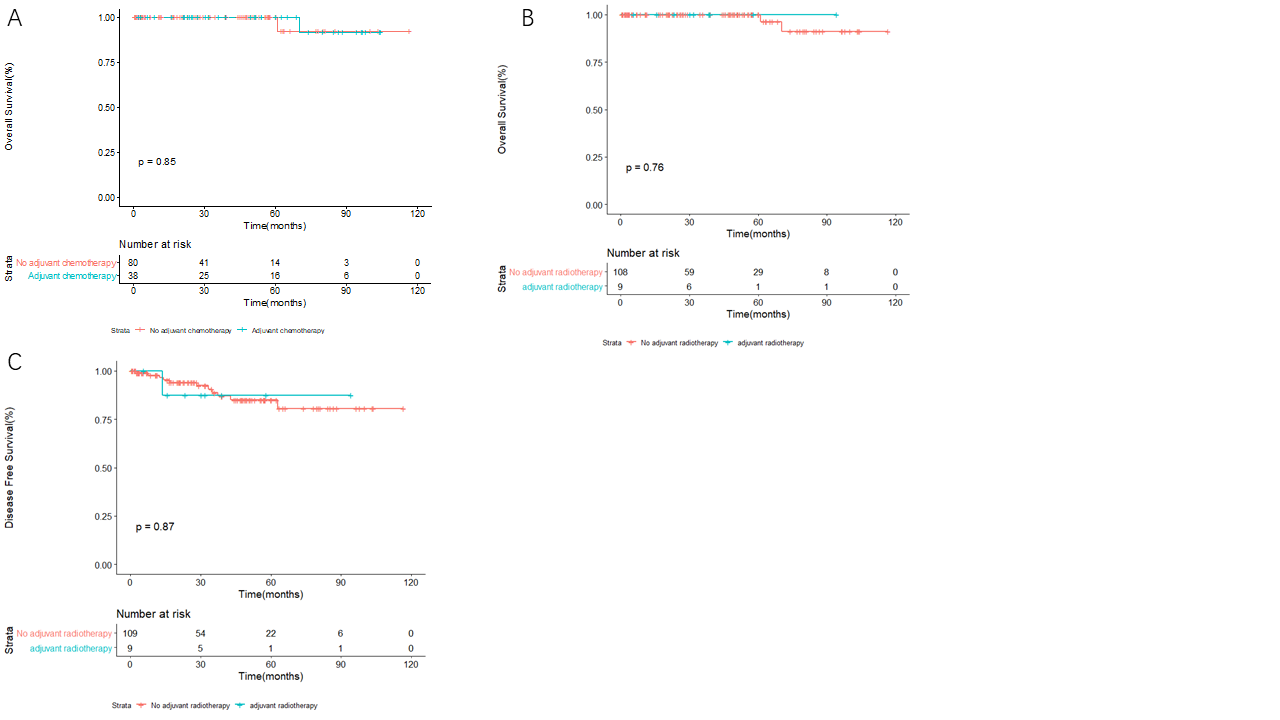

Supplement: Supplementary file 1 — Additional file 1. Figure S1: Kaplan–Meier survival analysis for OS in patients receiving adjuvant chemotherapy at stage I (A). DFS(B) and OS(C) in patients receiving adjuvant radiotherapy at stage I [file 12890_2022_2097_MOESM1_ESM.tif]

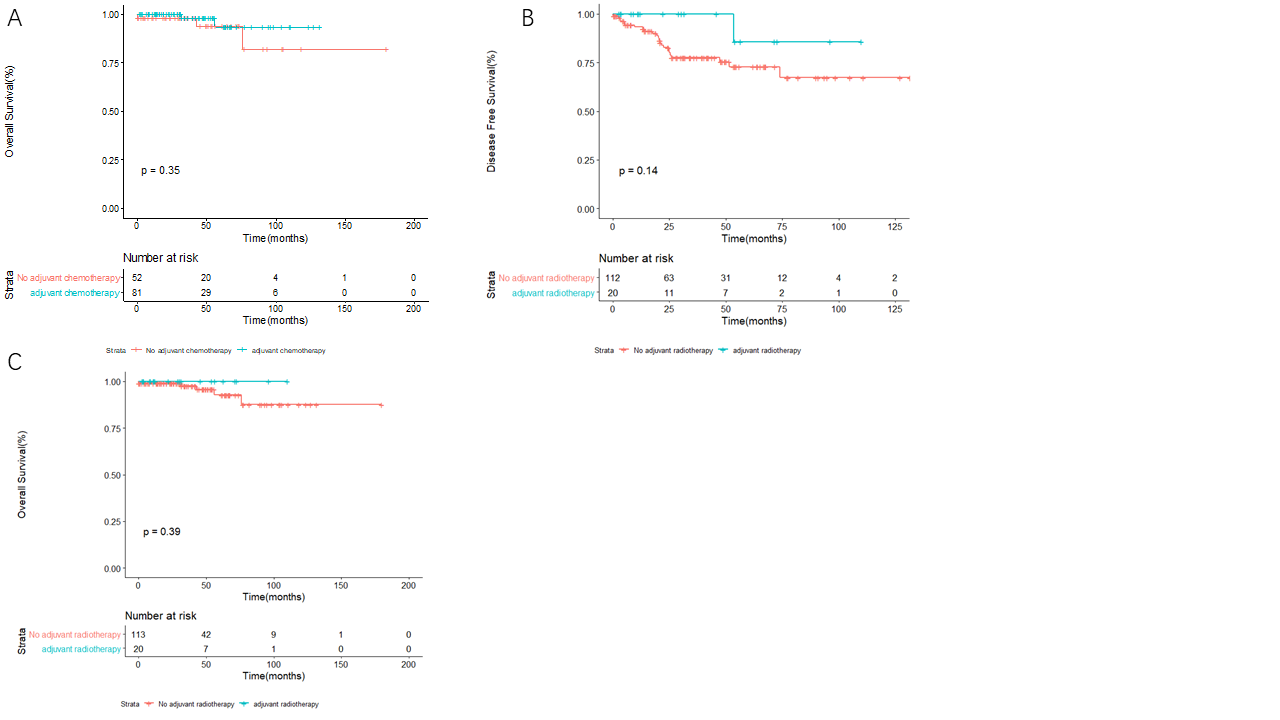

Supplement: Supplementary file 2 — Additional file 2. Figure S2: Kaplan–Meier survival analysis for OS in patients receiving adjuvant chemotherapy at stage II (A). DFS(B) and OS(C) in patients receiving adjuvant radiotherapy at stage II [file 12890_2022_2097_MOESM2_ESM.tif]

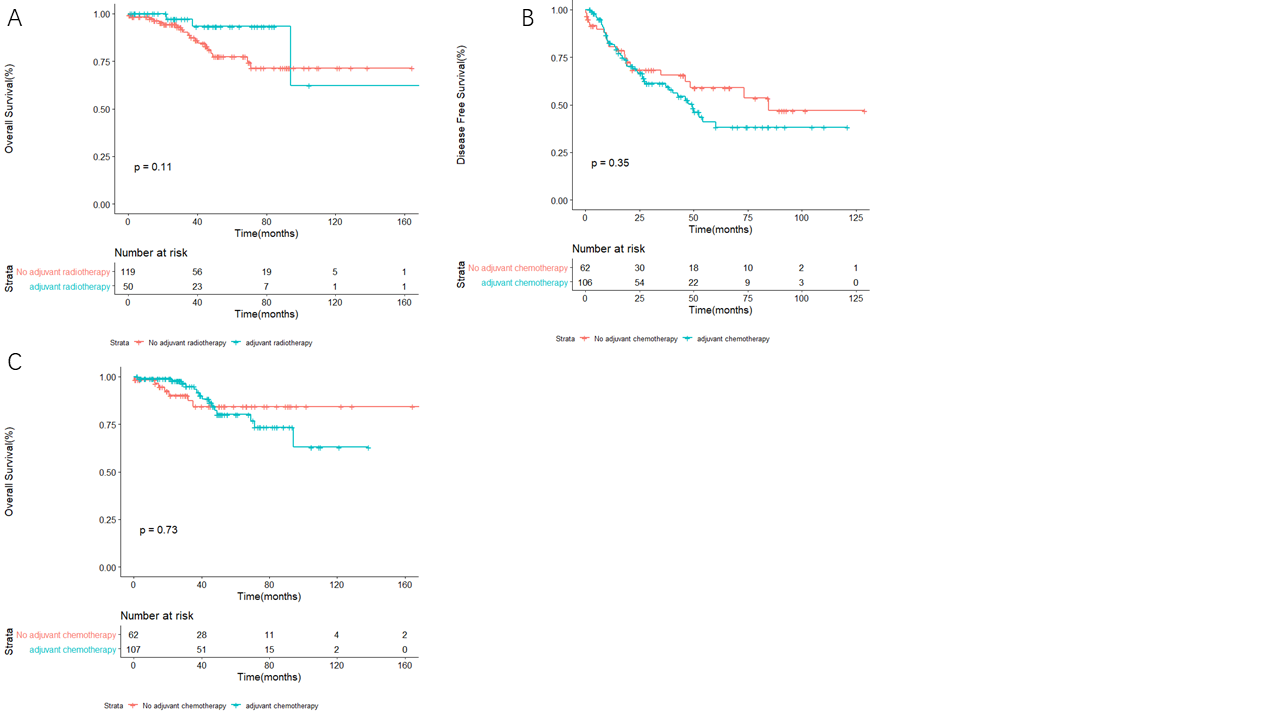

Supplement: Supplementary file 3 — Additional file 3 Supplement Figure 3 Kaplan–Meier survival analysis for OS in patients receiving adjuvant radiotherapy at stage III (A). DFS(B) and OS(C) in patients receiving adjuvant chemotherapy at stage III [file 12890_2022_2097_MOESM3_ESM.tif]

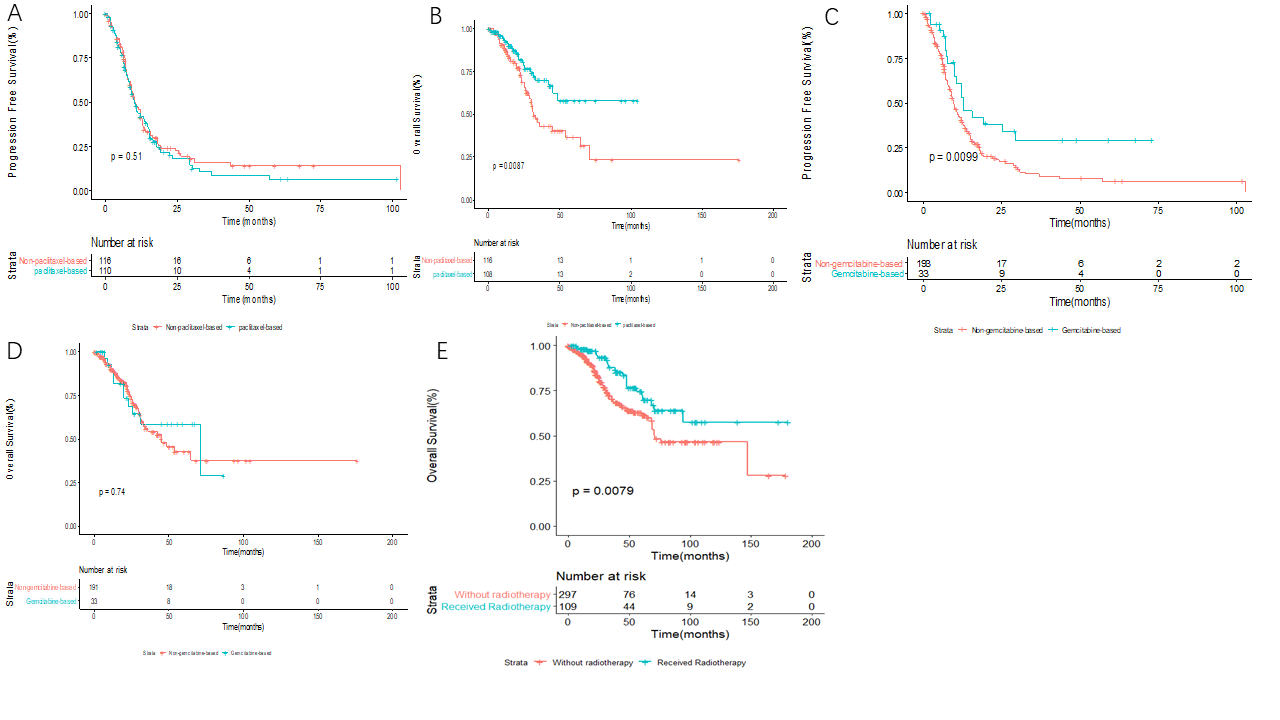

Supplement: Supplementary file 4 — Additional file 4. Figure S4: Kaplan–Meier survival analysis for PFS (A) and OS (B) in patients receiving paclitaxel-based chemotherapy as first-line regimen at stage IV or after relapsed. PFS (C) and OS (D) in patients receiving gemcitabine-based chemotherapy as first-line regimen at stage IV or after relapsed. OS in patients at stage IV or after relapsed receiving radiotherapy (E) [file 12890_2022_2097_MOESM4_ESM.tif]

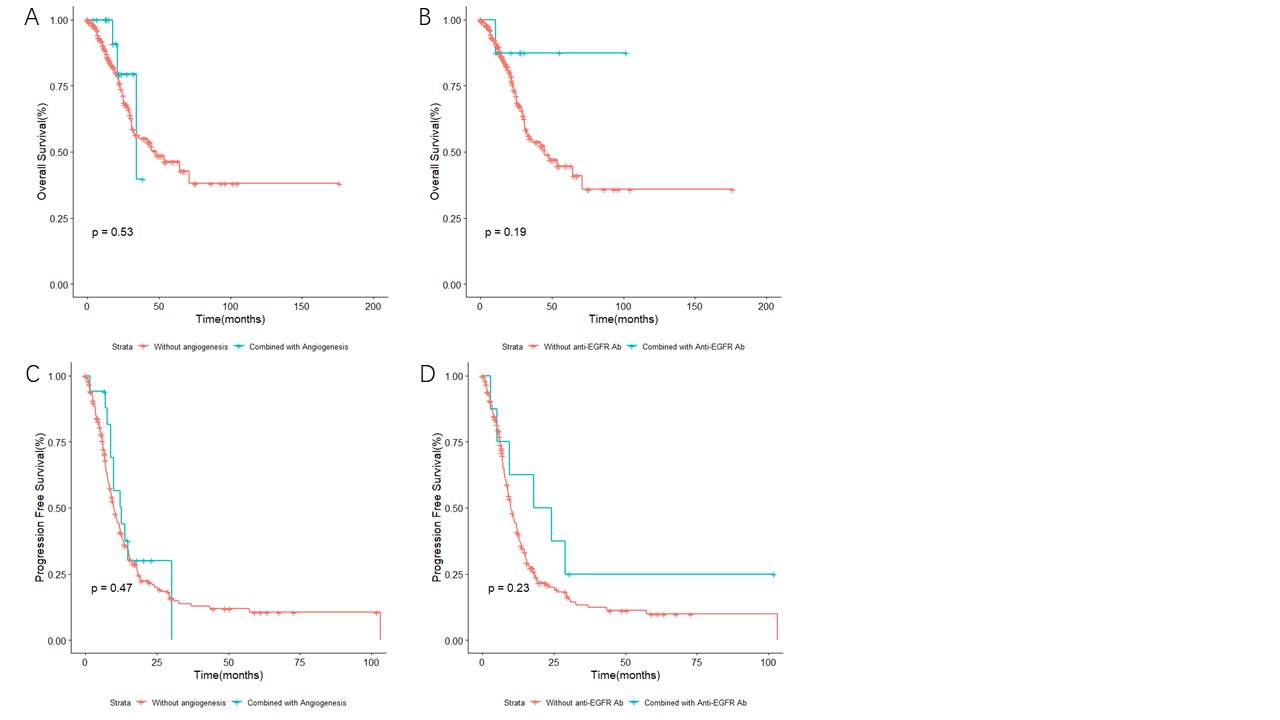

Supplement: Supplementary file 5 — Additional file 5. Figure S5: Kaplan–Meier survival analysis for OS in patients receiving anti- angiogenesis (A) and anti- EGFR (B) therapy at stage IV or after relapsed. Kaplan–Meier survival analysis for PFS in patients receiving anti-angiogenesis (C) and anti-EGFR (D) therapy at stage IV or after relapsed [file 12890_2022_2097_MOESM5_ESM.tif]

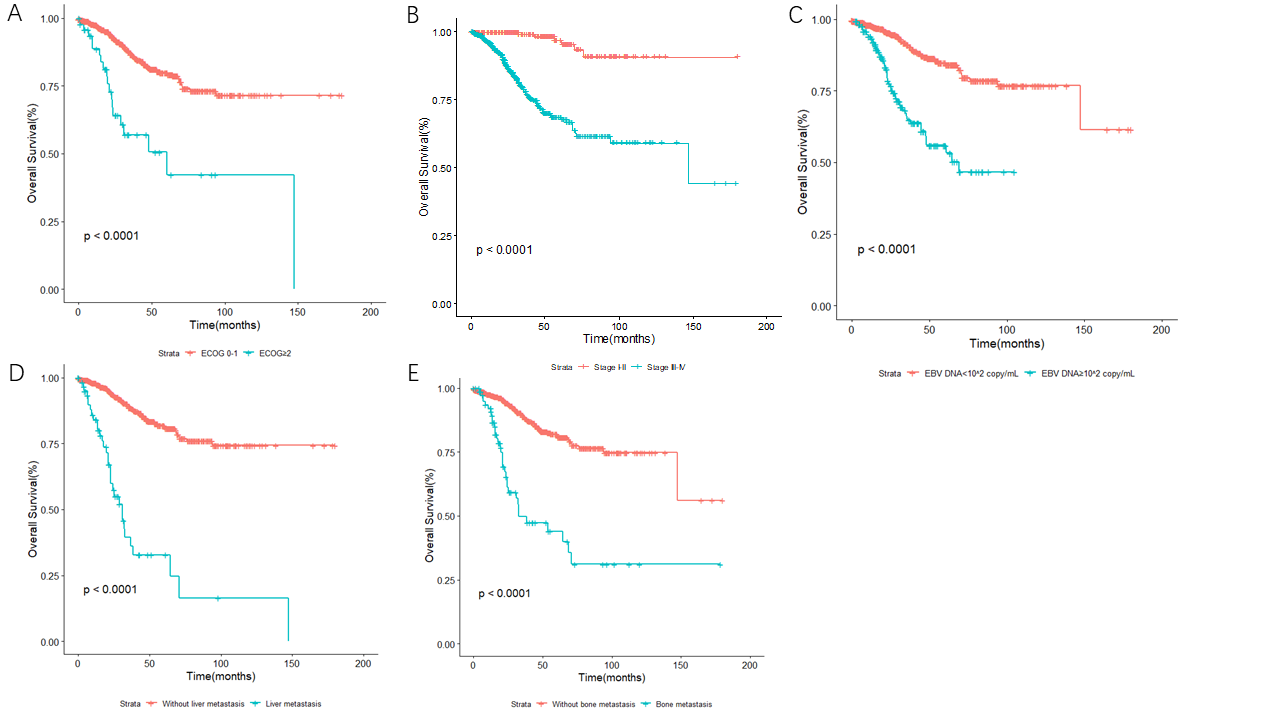

Supplement: Supplementary file 6 — Additional file 6. Figure S6: Kaplan–Meier survival analysis for OS according to ECOG PS≥2 (A), stage III-IV (B) , high level of EBV-DNA (C), liver metastasis (D) and bone metastasis (E) [file 12890_2022_2097_MOESM6_ESM.tif]
